# Evaluating research evidence for individualized treatment planning: the Clinician's Holistic Evidence Checklist (CHEC)

**DOI:** 10.3389/fpsyg.2026.1820872

**Published:** 2026-05-05

**Authors:** Ryan L. Davies, Julie C. Clark, Mahala Martin, Kylie Rice

**Affiliations:** School of Psychology, University of New England, Armidale, NSW, Australia

**Keywords:** clinical decision making, evidence based practice, evidence based treatment, intervention planning, treatment planning

## Introduction

The scientist-practitioner framework requires clinicians to appraise and apply evidence-based research to inform effective service delivery (Jones and Mehr, 2007). This approach aligns directly with evidence-based practice (EBP) which requires clinicians to systematically integrate three core components, commonly known as the *three-legged stool*, to inform decision-making and optimize outcomes (Spring, 2007). These three components of the EBP model include: best available research, clinical expertise, and client characteristics, culture and preferences (Lilienfeld et al., 2013; Spring, 2007). EBP has become increasingly embedded in mental health practice across allied health disciplines over the past few decades ([American Psychological Association (APA), 2006]; Melchert et al., 2024) and is fundamental within professional competency frameworks (Australian Health Practitioner Regulation Authority, 2024; Rice et al., 2025). As the analogy of the three-legged stool suggests, no single component should dominate clinical decision-making. Instead, clinicians must understand the relative strengths and limitations of each “leg” for an idiographic, deliberate, EBP approach to clinical decision-making (Melchert et al., 2024). This paper introduces the Clinician's Holistic Evidence Checklist (CHEC), a practical tool designed to support clinicians in integrating research evidence, clinical expertise, and client characteristics, culture, and preferences when developing individualized mental health treatment plans.

## Practical approaches to EBP

Spring (2007) explains how several attempts to improve EBP have been made, ranging from top-down to bottom-up approaches. Top-down approaches are nomothetic, providing guidelines or evidence-based treatment (EBT) lists that outline current research and make recommendations for the treatment of specific mental health disorders (for example, National Institute for Health and Care Excellence, (2026); The Royal Australian & New Zealand College of Psychiatrists, 2025). Bottom-up approaches are where clinical decisions are made based on the idiographic needs of the client relative to current research. Top-down approaches, alongside a large portion of the literature, emphasize the strength and efficacy of scientific evidence as a priority component of the EBP model (Lilienfeld et al., 2013). Still, holistic EBP demands that, in addition to efficacy, the context of client-centered, clinical utility is considered [American Psychological Association (APA), 2006] and clinical expertise evaluated (Satterfield et al., 2009). These three EBP components are congruent with many professional competency frameworks which stress the integration of scientist-practitioner, clinical assessment, intervention, and cultural responsiveness (Rice et al., 2025). However, critical appraisal of the literature is often emphasized at the expense of clinician expertise and client preference (Melnik et al., 2025; Satterfield et al., 2009). Moreover, whilst tertiary education programs teach the three EBP components, guidance about how to integrate the model holistically for practical and clinical relevance, particularly at the level of individual client decision-making, is largely absent (Jones and Mehr, 2007; Melnik et al., 2025).

Satterfield et al. (2009) discussed how different disciplines can approach EBP in various ways. First, in relation to best available research, psychology highlights empirically supported treatment, whereas social work and nursing assert that critical appraisal of the literature is dependent on the clinical question. Second, psychology and nursing underscore the importance of how client characteristics influence treatment outcome; however, social work expands on this to include the influence of environmental context. Finally, public health and social work emphasize the largely unaddressed influence of institutional factors and availability of resources. Satterfield et al. (2009) proposed the *Transdisciplinary Model of Evidence-Based Practice* which integrates multiple disciplinary perspectives to place systematic, contextualized decision-making at the center of EBP. This model, however, does not provide a structured or applied tool to guide clinicians through systematic decision-making in everyday clinical practice.

## Barriers to EBP

Although clinicians are required to adhere to EBP, numerous practical challenges hinder application (Ward et al., 2022). Barriers to the adoption of EBP include an overreliance on personal experience, misconceptions about EBP, misinterpretation of research findings, and practical or educational barriers (Lilienfeld et al., 2013). Additionally, there are significant gaps in understanding the fundamental principles of EBP, contributing to a lack of application and leading to the need for specific recommendations to equip clinicians with evidence appraisal skills (Melnik et al., 2025). Evidence appraisal within EBP involves a systematic evaluation of research to ascertain validity, reliability, and applicability to clinical practice, facilitating informed, evidence-based decisions relevant to specific clients and contexts (Straus et al., 2019). Although several tools support critical appraisal of the literature, clinicians are also prone to bias and must be reflective of personal limitations in knowledge and competence [American Psychological Association (APA), 2006; Rice et al., 2025]. Lilienfeld et al. (2013) discussed the concept of naïve realism, whereby clinicians overly rely on their own clinical judgement, increasing the risk of misinterpreting treatment effectiveness. Moreover, clinicians with less experience may not have awareness around personal limitations regarding case formulation and the integration of scientific evidence (Huisman and Kangas, 2018). Hence, clinically relevant practice tools which guide clinicians to reflect on their own expertise, limitations, and contextual constraints within idiosyncratic client presentations are required (Rousseau and Gunia, 2016).

## Existing EBP quality assessment

Currently, multiple tools support critical appraisal of research, such as [Joanna Briggs Institute (JBI), 2020], Mixed Methods Appraisal Tool (Hong et al., 2018), and the Grading of Recommendations Assessment, Development and Evaluation (GRADE) tool which provides a structured approach to evaluating and rating the certainty of evidence (Prasad, 2024). However, these tools primarily focus on one “leg” of the EBP model, that is, best available research. To extend appraisal beyond methodological quality and assess clinical applicability, Nguyen et al. (2021) created the VICORT checklist (Validity, Indication-informativeness, Clinical relevance, Originality, Risk–benefit comprehensiveness, Transposability). Several domains of the VICORT, such as validity and clinical relevance, are relevant across health disciplines. However, the VICORT is primarily designed for medical and epidemiological contexts. Domains such as indication-informativeness and originality reflect research and medical priorities and are less directly aligned with mental health treatment planning contexts, where client-centered EBP is needed. Importantly, VICORT does not facilitate clinician reflexivity, with the absence of domains such as clinical expertise, feasibility within wider environmental context, and client characteristics or preferences. This limits the practical utility of the VICORT for mental health clinicians.

The American Psychological Association (APA) (2021) developed *Guidelines on Evidence-Based Psychological Practice in Health Care* which comprehensively covers EBT alongside client populations and practice context. To support clinicians to work within an EBP framework, Melchert et al. (2024) proposed a decision aid flow chart aligned with the 12 American Psychological Association (APA) (2021) guidelines. However, the yes/no response style of this decision aid does not allow for depth of evaluation, and questions posed at the domain levels do not foster clinician reflexivity. As Stuart and Lilienfeld (2007) suggest, there is a clear need to be able to measure and evaluate EBP using objective criteria, to aid the integration of scientific evidence within the client and clinical contexts. Taken together, these limitations highlight a gap for a brief, clinician-facing tool that supports transparent integration of evidence quality, clinical feasibility, and client fit during routine treatment planning. Unlike existing appraisal tools that focus primarily on methodological quality or guideline adherence, the CHEC explicitly integrates evidence strength, client applicability, and clinician feasibility into a single structured framework to support individualized treatment planning.

## The Clinician's Holistic Evidence Checklist (CHEC)

**Figure 1:**
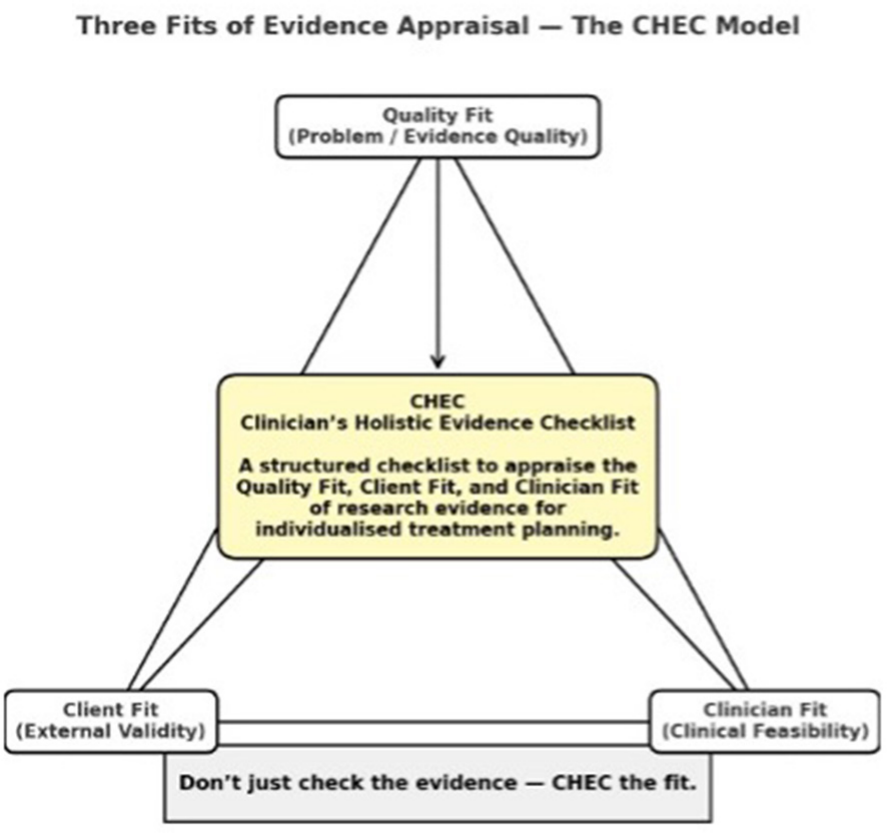
Clinician's Holistic Evidence Checklist (CHEC): a structured checklist to appraise the Quality Fit, Client Fit, and Clinician Fit of research evidence for individualized treatment planning.

The Clinician's Holistic Evidence Checklist (CHEC) is a practical tool designed to support clinicians across health disciplines and operationalize the three components of EBP for individualized treatment planning in mental health care settings. Grounded in the transdisciplinary model of EBP (Satterfield et al., 2009), the CHEC supports scientist-practitioner decision-making and clinician reflexivity by integrating three fits of evidence appraisal that must be considered together when planning individualized care (Figure 1).

The CHEC was developed through an iterative, theory-informed process. First, the research team reviewed established models of evidence-based practice and existing critical appraisal frameworks across health science to identify core domains relevant to clinical decision-making. This review informed the initial item pool, structured around appraisal of evidence quality, client applicability, and clinical feasibility. The draft CHEC was then circulated for expert review to a multidisciplinary panel comprising five research experts with experience in evidence synthesis and methodology, and five practicing clinicians with expertise in mental health service delivery. Feedback focused on clarity, clinical relevance, feasibility, and alignment with real-world practice. The research team reviewed all feedback and refined items through consensus discussion, resulting in the final CHEC framework and scoring approach presented here.

First, *Quality Fit* (Internal Validity) evaluates whether the available evidence for an intervention is methodologically sound and trustworthy to inform clinical decision-making. Second, *Clinician Fit* (Clinical Feasibility) fosters reflective practice by prompting clinicians to evaluate whether they can competently, ethically and realistically deliver the intervention within their service context and scope of practice. Third, *Client Fit* (External Validity) considers whether the intervention is applicable to the individual client's clinical presentation, identity, cultural context, values, and preferences. The full CHEC, which guides clinicians through structured appraisals of Quality Fit, Clinician Fit, and Client Fit, and the scoring guidance can be found in the Supplementary File 1.

In Parts A to C of the CHEC, clinicians rate each criterion using a five-point Likert scale. Part A is applied to the included studies or body of evidence for a given intervention, whereas Parts B and C are completed holistically, considering the whole evidence base in relation to a specific client and clinician context. In Part D, clinicians integrate these ratings to formulate an overall confidence judgement, supporting individualized mental health treatment planning. Part D scoring is intended to support a brief overall confidence judgement (e.g., low/moderate/high) and to document the clinician's rationale, rather than to produce a strict cut-off score. The scoring thresholds provide structured guidance rather than rigid decision rules, with a final judgement requiring clinical interpretation. The CHEC is not intended to replace formal evidence synthesis or guideline development processes. Rather, it provides a pragmatic framework to support clinicians in translating existing research evidence into contextually informed, client-centered clinical decisions. Importantly, the CHEC is designed to support integration of evidence even when the three domains are not fully aligned. In practice, clinicians may encounter situations where evidence quality is strong, but client fit or clinician feasibility is limited, or vice versa. In these cases, the CHEC does not prescribe a single “correct” decision, but instead structures transparent clinical reasoning by making trade-offs explicit. Quality Fit provides an upper bound on confidence in the evidence, while Client and Clinician Fit determine whether that evidence can be meaningfully and appropriately applied in a given context. This supports deliberate, context-sensitive decision-making consistent with evidence-based practice.

## Clinical implications

The CHEC extends existing appraisal tools and guidelines by explicitly incorporating client preferences and clinician expertise, thus facilitating holistic and deliberate EBP for individual, mental health treatment planning. By integrating three fits of evidence appraisal, in line with the EBP model (Satterfield et al., 2009), the CHEC supports mental health clinicians to execute core professional competencies, including: ethical decision-making, cultural responsiveness, and reflexivity (Rice et al., 2025). In contrast to appraisal tools that focus primarily on evidence quality or guideline adherence, the CHEC is designed to explicitly document how research evidence, clinical expertise, and client preferences are integrated in individual treatment planning. By making these judgements explicit, the CHEC supports consistency and transparency across clinicians and settings and provides a shared language for supervision and client discussions. This structured approach supports clinicians to make transparent decisions even when evidence quality, client characteristics, and feasibility considerations are not fully aligned.

The CHEC uses clinically relevant language to support mental health clinicians to bridge the scientist-practitioner divide, with practical application across health disciplines and for tertiary education. In comparison, alternative appraisal tools come from an academic lens (for example JBI, GRADE) or are contextually specific (for example, VICORT). In mental health clinical settings, the CHEC facilitates a systematic appraisal of research evidence, reflexivity and ensures that clinical decisions align with best-practice standards. In one study, 80% of psychologists agreed on the importance of the evidence-base to inform case formulation (Thrower et al., 2024), which specifically requires integration of EBP (Huisman and Kangas, 2018). The CHEC is designed to support this process by providing a clearly documented rationale for the treatment plan. Furthermore, applying the evidence-base to inform collaborative decision-making with clients is a complex and fundamental step in the EBP process that can be overlooked (Spring and Neville, 2011). The CHEC tool can aid collaborative discussion with clients and facilitate informed consent for treatment plans. In supervision and tertiary education, the CHEC is a practical tool which steps trainees through how to evaluate the three components of EBP and facilitates the integration of this knowledge to holistically inform individualized treatment planning. Future work should examine the CHEC's usability in clinical and training contexts, and the consistency of ratings across clinicians and supervisory settings.

## Conclusion

The CHEC integrates all three EBP components through evaluating Quality Fit, Client Fit, and Clinician Fit; providing a client-centered, practical checklist that is grounded in theory (Satterfield et al., 2009; Spring, 2007). Understanding and evaluating the best evidence-based research is a critical component of EBP; however, for practical application that informs individualized mental health treatment plans it is imperative to integrate clinical expertise and client characteristics into decision-making. The CHEC extends the literature and existing critical appraisal tools to operationalize EBP for mental health clinicians. Because the CHEC is explicitly designed to be contextually relevant, it aligns with the transdisciplinary model of EBP (Satterfield et al., 2009), bridging disciplinary perspectives within mental health practice and supporting more integrated and coherent application of evidence-based decision-making across settings. The CHEC may help address existing barriers to EBP, with practical implications for mental health clinical practice across health disciplines and for teaching integrated, holistic EBP in tertiary education. In educational contexts, the CHEC provides a structured framework to teach, scaffold, and evaluate the development of evidence-based decision-making skills among trainees. Future research may examine the CHEC's utility in clinical research projects, supervision, and training evaluations, including its role in enhancing transparency and consistency in treatment planning decisions.
