# Supplementary material for: Evaluating research evidence for individualized treatment planning: the Clinician's Holistic Evidence Checklist (CHEC)
Source: Front Psychol. 2026 May 5;17:1820872. doi: 10.3389/fpsyg.2026.1820872 (PMC13183807; doi:10.3389/fpsyg.2026.1820872)
Supplement: Supplementary file 1 [file Table_1.docx]

**CHEC Explanation Companion Document**

**Part A. Study Quality (Internal Validity)**

This section checks if the selected studies were designed and reported well enough to give you confidence in the findings.

| **1. Study Design**  **Is the study design appropriate for testing whether the intervention works?** |
| --- |

**Where to Look:**

| **In a systematic review:**   - **Methods section → “Eligibility Criteria” or “Types of Studies Included”**   - What study designs were eligible? - **Results section → Characteristics of Included Studies**   - What designs were actually included? |
| --- |

| **In a single study:**   - **Methods section → “Study Design” or first paragraph of Methods**   - Authors usually state the design explicitly. - Look for phrases such as:   - *Randomised controlled trial (RCT)*   - *Randomised clinical trial*   - *Controlled trial*   - *Quasi-experimental*   - *Pre–post design*   - *Cohort study*   - *Case-control study*   - *Cross-sectional study* - Check whether:   - Participants were **randomly allocated** to groups.   - There was a **comparison or control group**.   - The study followed participants over time (longitudinal) or measured outcomes at one time point only. - If the design is unclear, review:   - The **participant flow diagram**   - The **“Procedure” section**   - How the groups are described in the Results |
| --- |

***Note:*** If a study does not clearly describe how participants were allocated or whether there was a comparison group, the design is likely weaker for establishing intervention effectiveness.

| **2. Bias Control**  **Were steps taken to minimise bias and improve fairness?** |
| --- |

**Where to look:**

| ***Systematic Review*** | |
| --- | --- |
| - Were the included studies mostly at a low risk of bias? - Did the authors explain how risk of bias affects confidence in the results? | - Go to the Risk of Bias table/figure (often in the Results section). - Look for an overall summary. Are most studies judged “low risk,” or are many “high/unclear risk” - Check if the authors explain how risk of bias might affect confidence in the results. |

| ***Single Study*** | |
| --- | --- |
| If an RCT:   - was **randomisation**done appropriately?     If a non-randomised study:     - Were important **confounders**  measured and controlled for?     For all study designs:   - Was there a **comparator/control group** and were groups similar at baseline? - Were all**planned outcomes** reported (i.e., no evidence of cherry-picking)? | - **Randomisation:**Methods - look for a description of how participants were randomised (e.g., random number generator, sealed envelopes).      - **Confounders:**Methods**-**look for whether key confounders (e.g., age, gender, geographic location, illness severity) were measured and adjusted for in the analysis.      - **Comparator:**Study Design - Was there a comparator or control group? If so, check baseline demographics to ensure it was comparable to the intervention group. - **Planned Outcomes:** Results - confirm that all pre-stated outcomes were reported (compare to trial registration/ protocol/ hypotheses). |

| **3. Sample Size & Retention**  **Was there adequate sample size and participant retention?** |
| --- |

**Where to look:**

| ***Systematic Review*** | |
| --- | --- |
| - Were included studies generally large enough to provide reliable results? - Did most studies retain participants, or were high dropout rates common? | **Sample Size**   - **Study characteristics table** (usually in Results or Appendix): check the number of participants in each included study. - **Risk of Bias/Limitations:**Look for comments on small samples     **Retention**   - **Risk of Bias/Limitations**: see if the authors note whether studies had “high attrition.” - **Results/Discussion**: look for mention of dropout rates across studies and whether this affected conclusions. |

| ***Single Study*** | |
| --- | --- |
| - Did the study justify its sample size (e.g., with a power calculation), and was the planned sample size achieved? - Was retention adequate (dropout < 20%), if not was there a reasonable explanation | **Sample Size**   - **Methods (participants):** check how many participants were recruited, whether a sample size/power calculation was reported, and how many participants were planned vs actually included. - **Discussion:** check if authors acknowledge small samples as limitations.     **Retention**   - **Results:** review the participant flow diagram (often CONSORT-style) to see how many dropped out and whether reasons were provided. - **Discussion:** check if authors acknowledge high dropout as limitations. |

| **4. Outcome Measures**  **Were outcomes measured in a way that is accurate and appropriate for the construct?** |
| --- |

**Where to look:**

| ***Systematic Review*** | |
| --- | --- |
| - Were **recognised** psychometric  **instruments**(for psychological outcomes), and/or standardised, accepted measurement procedures (e.g., physiological measures) used? - Did the outcome measures match what the study claimed to measure? | **Recognised Measures**   - **Tables of included studies:**check what tools/measures were used. - **Methods/Results sections:**see whether the review comments on whether studies used validated or consistent measures. - **Risk of bias/quality assessment:** reviewers may note if outcomes were poorly defined or selectively reported. |

| **5. Reporting Transparency**  **Were the study methods and results reported in a way that allows you to trust and use the findings?** |
| --- |

**Where to look:**

| ***Systematic Review*** | |
| --- | --- |
| - Were results reported with **effect sizes** and **confidence intervals** (not just “significant / not significant”)? - Was **missing data** from included studies acknowledged and handled (e.g., sensitivity analyses)? - Was the review **preregistered** (e.g., PROSPERO) and protocol followed? - Were **funding sources** and **conflicts of interest** disclosed? - Did the authors state adherence to **PRISMA guidelines**? | - **Effect Size:** Results - “Results of individual studies,” forest plots, effect size tables. - **Missing Data:**Methods/Results: “Risk of bias,” “Synthesis methods,” “Sensitivity analyses.” - **Registration:** Methods - “Protocol and registration” (PROSPERO/CRD). - **Declarations:**funding and conflicts of interests. - **Reporting Guidelines:**Appendix/ supplemental file - PRISMA statement/checklist. |

| ***Single Study*** | |
| --- | --- |
| - Were results reported with **effect sizes**and **confidence intervals** (clinical significance) not just p-values)? - Was **missing data** explained and handled to reduce bias (e.g., imputation, sensitivity analyses)? - Was the study **preregistered** (trial registry or protocol available)? - Were **funding sources** and **conflicts of interest** declared? - Did reporting follow recognised **guidelines**? | - **Effect Size:** Results – look if the study reports the difference between the intervention and comparison group with confidence intervals (CIs). If the CI for a mean difference includes zero, the study cannot rule out the possibility of no real effect, making the finding less trustworthy. If the study does not report confidence intervals and only provides mean differences or p-values, this is a concern because you cannot judge the precision or reliability of the effect. - **Missing Data:**Methods - “Statistical analysis” (missing data handling). - **Registration:**Methods - “Trial registration/protocol.” - **Declarations:**funding, conflicts of interest. - **Reporting Guidelines:**Appendix/ supplemental file - CONSORT/ for RCTs, STROBE for observational |

| **6. Consistency of Findings** |
| --- |

**Where to look:**

| **Systematic Reviews** |
| --- |
| Systematic Review with Meta-Analysis:   - Look at **forest plot -**are most of the results pointing to the same side of the line of no effect? - Check the **heterogeneity statistic (I²)** → below ~40% = generally consistent; above 75% = results vary a lot. - See if the authors provide **subgroup analyses or explanations** for differences.     Systematic Review without Meta-Analysis:   - Review the results section to see whether studies generally point in the same direction. - Check whether the authors comment on consistency or provide explanations for differing results (e.g., differences in population, setting, intervention intensity, outcome measures). - Look for patterns across studies (e.g., “most studies found improvements in X,” “findings were mixed,” “only one study found an effect”). |

| **Single Study** |
| --- |
| - Compare results sections across the studies you’re reviewing. Do they generally support or contradict each other? |
