# Supplementary material for: Evaluating research evidence for individualized treatment planning: the Clinician's Holistic Evidence Checklist (CHEC)
Source: Front Psychol. 2026 May 5;17:1820872. doi: 10.3389/fpsyg.2026.1820872 (PMC13183807; doi:10.3389/fpsyg.2026.1820872)
Supplement: Supplementary file 2 [file Table_2.docx]

**Part A. Study Quality (Internal Validity)**

This section checks if the selected studies were designed and reported well enough to give you confidence in the findings.


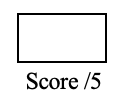


**1. Is the study design appropriate for testing whether the intervention works?**

*This question checks whether the study design allows you to judge if the intervention actually caused the outcome. Designs with appropriate comparison groups provide stronger evidence than those that rely on observation alone.*

**Score: (1)** **Weak:** Case studies, expert opinion, narrative reviews; **(2)** **Moderate:** Observational studies e.g., cohort, case-control, cross-sectional; **(3)** **Strong:** Non-randomised intervention studies e.g., quasi-experimental, single-group pre-post, natural experiments; **(4)** **Very Strong:** Randomised Control Trials; **(5)** **Best:** High-quality systematic review of Randomised Control Trials.


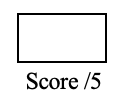


**2. Were steps taken to minimise bias and improve fairness?**

*This question checks whether the study design and reporting reduce the risk of misleading results, so observed outcomes can  be attributed to the intervention rather than methodological flaws.*

**Score:** **(1)** High risk of bias – major methodological problems; **(3)** Some risk of bias – acceptable but important limitations; **(5)** Low risk of bias – strong methods that minimise bias.


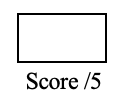


**3. Was there adequate sample size and participant retention?**

*This question checks whether the study had enough participants, and kept enough of them, to produce reliable findings. Small or underpowered studies, or those with high dropout, may give unstable or biased results, while larger, well-retained samples provide stronger and more trustworthy evidence.*

**Score:** **(1)** Very small sample, high attrition; **(3)** Moderate sample, some attrition; **(5)** Large, well-justified sample with good retention.


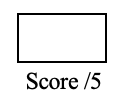


**4. Were outcomes measured in a way that is  appropriate for the construct?**

*This question checks whether the study used outcome measures that appropriately capture what the intervention was intended to change. Using appropriate measures increases confidence that the results meaningfully reflect the construct of interest. Poorly defined, ad-hoc, or mismatched measures reduce confidence in the findings.*

**Score: (1)** Measures unclear, inappropriate, or poorly justified; **(3)** Measures somewhat appropriate but with limitations; **(5)** Measures clearly justified and widely recognised as accurate for the construct.


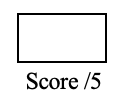


**5. Were the methods and results transparently reported in a way that allows you to trust the findings?**

*This question looks at whether the studies were reported clearly and honestly. Transparent reporting, such as showing effect sizes with confidence intervals, handling missing data, preregistering protocols, and declaring funding or conflicts, helps you judge if the findings are trustworthy.*

**Score:** **(1)** Major flaws, results not trustworthy; **(3)** Some issues but usable with caution; **(5)** High-quality analysis and transparent reporting.


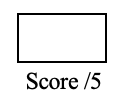


**6. Do results from multiple studies consistently point in the same direction?**

*This question looks at whether there are consistent findings in the literature. When findings are consistent, we can be more confident that the effect is real rather than due to chance or context. If studies conflict without clear explanation, certainty is reduced because it is unclear whether the intervention will work reliably in practice.*

- Consistent findings = higher certainty.
- Mixed findings may be explainable (e.g., setting, population).
- Conflicting findings with no explanation = low certainty.

**Score: (1)** Highly inconsistent studies contradict each other, no clear explanation; **(3)** Some variation differences exist but partly explained by context/population; **(5)** Consistent most studies agree, effects point in the same direction.

**
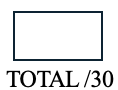
**

| **PART A SCORE** |
| --- |

**Part B. Clinician Fit (Clinical Feasibility)**

This section checks whether the intervention, based on the body of evidence you reviewed in Part A, is feasible for you to deliver in your practice context. Even if the evidence is strong and fits the client, it must also align with your skills, training, resources, service constraints, and ethical competence. These items prompt reflexivity about whether you can realistically and safely implement the intervention in your setting.


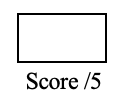


**7. Competence**

**Do you have an appropriate combination of training, skills and knowledge, experience and/or availability of supervision to deliver this intervention as intended?**

**Score:** **(1)** Not within competence; **(3)**Somewhat aligned - would need to utilise supervision/additional support; **(5)** Fully competent and supported.


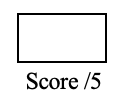


**8. Practical Feasibility**

**Can you realistically deliver this intervention in your practice?**

Consider time demands, session length, caseload pressures, resources, technology, and cost.

**Score:** **(1)** Not feasible; **(3)**Manageable with adaptation; **(5)** Easily feasible.


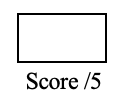


**9. Context Match**

**Were the conditions under which the intervention was tested similar to the context in which you would deliver it?**

e.g., individual vs group, online vs in-person, private vs community

**Score: (1)** Very different from your practice context; **(3)**Somewhat similar, but important differences exist; **(5)** Strong match to your practice context


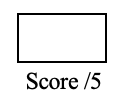


**10. Theoretical Orientation**

**Does this intervention align with your therapeutic approach?**

e.g., will it require major departure/adaptation from your usual practice? If so, evaluate motivation and/or level of dissonance this would create.

**Score:** **(1)** Not aligned, would require significant shift; **(3)**Somewhat aligned, some adaptation required; **(5)** Well aligned with your approach.


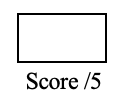


| **11. Ethical and Inclusive Practice**  **Can you delivery this intervention ethically in a way that is respectful to your client’s individual diversity?**   - Do I have the knowledge, training, or supervision support needed for this context? - Could aspects of this approach risk harm, distress, or value conflict for my client? - Does my approach maintain the client’s dignity, autonomy, and right to self-determination |
| --- |

*NB: Individual diversity* *includes, but is not limited to age, race, ethnicity, language, culture, immigration status, gender, gender identity and expression, sexual orientation, physical attributes, ability, disability, cognitive capacity, social and/or economic status, geographic location, educational attainment, and religious and/or spiritual orientation*

**Score:** **(1)** Concerns about safety/appropriateness; **(3)** Adequate with support; **(5)** Strong ethically sound and inclusive practice.


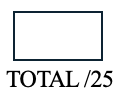
**PART B SCORE**

**Part C. Client Fit (External Validity)**

This section looks at how well the body of evidence you reviewed in Part A aligns with your client’s characteristics, culture, values, and preferences. Even the most rigorous study may not apply if the participants, cultural context, or outcomes differ from your client’s situation. Here, you consider the studies holistically, across the evidence you appraised in Part A, and judge how relevant and meaningful the findings are for this individual client in their real-world context.


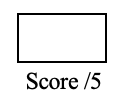


| **12. Identity and Individual Diversity**  **Was the individual diversity of your client represented in the study populations?** |
| --- |

*NB: Individual diversity* *includes, but is not limited to age, race, ethnicity, language, culture, immigration status, gender, gender identity and expression, sexual orientation, physical attributes, ability, disability, cognitive capacity, social and/or economic status, geographic location, educational attainment, and religious and/or spiritual orientation*

**Score:** **(1)** Very limited representation of clients with similar identity and lived experience characteristics; **(3)** Some representation, but key characteristics underrepresented; **(5)** Strong representation of clients with similar identity and lived experience characteristics.


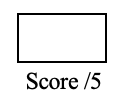


| **13. Were the study participants clinical presentation similar to your client in terms of diagnosis, symptoms severity and comorbidly?**   - **Diagnosis:** Was the diagnostic criteria used in the evidence similar to your client’s presentation - **Severity:** Were participants mild, moderate, or severe? Were crisis or high-risk clients included or excluded? - **Comorbidity:** Did the study include co-occurring presentation relevant to your client (e.g., substance use, trauma, neurodivergence) or exclude them? |
| --- |

**Score: (1)** Poor Fit- participants were very different from your client’s clinical presentation; **(3)** Partial Fit - some overlap, but some differences compared to your client’s presentation; **(5)** Strong Fit - participants were very similar to your client’s clinical presentation.


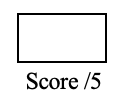


| **14. Did the study measure outcomes meaningful to your client?**   - Did the available evidence measure outcomes beyond symptom reduction, which are relevant to your client’s goals (e.g., functioning, wellbeing, relationship quality or quality of life)? |
| --- |

**Score:** **(1)** Highly irrelevant outcomes; **(3)** Somewhat relevant outcomes; **(5)** Highly relevant outcomes.


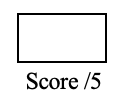


| **15. Is the intervention an acceptable fit with your client’s expressed preferences and values?**  Think about your client’s stated preferences and values:   - Have they expressed a preference for (or against) certain approaches (e.g., talking therapy vs. skill-based group vs. individual, medication vs. non-medication?) - Is the format, intensity, and mode of delivery acceptable to them (e.g., online vs. face-to-face, length and number of sessions, homework expectations)? - Do they view the intervention as relevant and aligned with their goals? |
| --- |

**Score: (1)** Poor fit - intervention conflicts with client’s stated preferences or is unlikely to be acceptable; **(3)** Partial fit - intervention partly aligns, but some adaptations may be needed; **(5)** Strong fit - intervention closely matches the client’s preferences and goals.

**
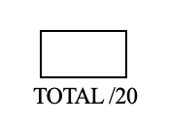
**

| **Part C Score** |
| --- |

**Part D. CHEC the FIT - Confidence Rating**

**Step 1. Score Study Quality (Part A)**

| **If using multiple primary studies:** | **If using a Systematic Review** | **If using a single primary study:** |
| --- | --- | --- |
| 1. Score Q 1-5 for each study (max 25 per study). 2. Average the study scores. 3. Add Q6 Consistency.   **Final Part A range = 5–30** | 1. Score Q1–5 based on the quality of the included primary studies (not the review methods). 2. Score Q6 Consistency.   **Final Part A range = 5–30** | 1. Score Q1–5 only. 2. Q6 = 0 (consistency cannot be assessed).   **Final Part A range 5-25** |

**Step 2. Apply the Part A Gatekeeper Rule**

- If more than half of studies scored “1” on **(Q1)** **Study Design** OR **(Q2)** **Bias Control**, then overall FIT must be rated **Low Confidence** (stop here).
- If not → proceed to Step 3.

**Step 3. Score Clinician Fit (Part B) and Client Fit (Part C)**

Score these domains based on the **body of evidence as a whole**, not individual papers.

- Part B range: **5–25**
- Part C range: **5–20**

**Step 4. Classify Each Fit Domain**

|  | **Low** | **Moderate** | **High** |
| --- | --- | --- | --- |
| **Part A** | **≤15** | **16-25** | **26-30** |
| **Part B** | **≤12** | **13–20** | **21–25** |
| **Part C** | **≤10** | **11-16** | **17-20** |

**Step 5. Determine Overall Fit**

| Part A | Both Parts B and C = Moderate or High | Either Part B or C = Low | Both Parts B and C = Low |
| --- | --- | --- | --- |
| High | High Confidence | Moderate Confidence | Low Confidence |
| Moderate | Moderate Confidence | Moderate Confidence | Low Confidence |
| Low | Low Confidence | Low Confidence | Low Confidence |

**Step 6: Narrative Interpretation of Overall Confidence**

- **High Confidence:** There is a high level of confidence that the intervention is effective and can be appropriately delivered by this clinician to this client within the current context.
- **Moderate Confidence:** There is reasonable evidence of effectiveness, but important limitations exist in study quality, clinician feasibility, or client applicability. Clinical judgement, adaptation, and monitoring are advised.
- **Low Confidence:** There is insufficient confidence that the intervention can be effectively and appropriately delivered in this context due to critical methodological limitations, poor applicability to the client, or feasibility concerns.
